# Case report: Recovery of long-term delayed complete atrioventricular block after minimally invasive transthoracic closure of ventricular septal defect

**DOI:** 10.3389/fcvm.2023.1226139

**Published:** 2023-07-25

**Authors:** Jin Lu, Xingchen Lian, Ping Wen, Yuhang Liu

**Affiliations:** Department of Cardiovascular Surgery, Dalian Women and Children's Medical Group, Dalian, China

**Keywords:** ventricular septal defect, minimally invasive transthoracic occlusion, complete atrioventricular block, occluder, rhythm

## Abstract

**Introduction:**

Long-term delayed complete atrioventricular block (CAVB) is a serious complication of ventricular septal defect (VSD) closure treatment. Thus, cardiac surgeons have made significant efforts to explore its causes and reduce its incidence. In recent years, minimally invasive transthoracic closure (MITC) of VSD has been used widely and successfully in China as it is easy to repeat, ensures individualized closure, and can be debugged repeatedly. Theoretically, the possibility of the recurrence of CAVB is lower than that with transcatheter closure. Although the incidence of CAVB after MITC of VSD is inevitable, long-term delayed CAVB has rarely been reported.

**Case description:**

Herein, we report a case of delayed CAVB that occurred 2 years and 5 months after performing MITC of a perimembranous VSD. The cardiac rhythm recovered after the occluder was removed surgically.

**Conclusion:**

The findings of our case report emphasize that since delayed CAVB may occur in the long term after MITC of VSD, the safety of MITC of VSD should be reassessed, the indications for MITC should be strictly followed, and long-term follow-up, including lifelong follow-up, is recommended for patients postoperatively. In addition, the occluder should be removed surgically in patients with CAVB as it may restore normal heart rhythm.

## 1. Introduction

In recent years, cardiac surgeons have performed minimally invasive transthoracic closure (MITC) of the ventricular septal defect (VSD) in patients with complete atrioventricular block (CAVB) by combining the technical characteristics of cardiopulmonary bypass surgery and percutaneous transcatheter closure. The simplicity and ease of repeating the surgery, individualized closure, the lower age limit, and fewer complications (especially arrhythmia) have resulted in therapeutic effects that are superior to those of transcatheter closure (1, 2). CAVB cannot be avoided completely after MITC; however, few studies have reported the incidence of delayed CAVB (2–5). Herein, we report a case of delayed CAVB that occurred 2 years and 5 months after performing MITC of perimembranous VSD (pmVSD). The cardiac rhythm was found to recover after the surgical removal of the occluder. This case report presents the longest duration till the occurrence of CAVB and postoperative recovery of heart rhythm after MITC of pmVSD (5, 6). Through this case report, clinicians are reminded to pay attention to the safety of this surgery in young children.

## 2. Case description

The patient was a 7-month-old boy (weight: 7 kg) who presented to the hospital with sweating, shortness of breath, and growth retardation. Physical examination revealed that the heart sounds were strong and rhythmic. Systolic murmurs were indicated in L3–4, and P2 was not hyperactive. Echocardiography revealed enlargement of the left atrium and left ventricle and a pmVSD of 5 mm. Chest radiography revealed congestive changes. Thus, a diagnosis of pmVSD was made.

In January 2017, MITC of pmVSD was performed under esophageal ultrasound guidance at our hospital. A No. 4 equilateral closure umbrella was placed during the surgery, with the double umbrella facing the opened well. The push-and-pull test was reliable, and the closure umbrella was released successfully. Residual shunt, arrhythmia, or abnormal recovery was not observed postoperatively. The child was in good condition after discharge and was followed up regularly.

**Figure 1:**
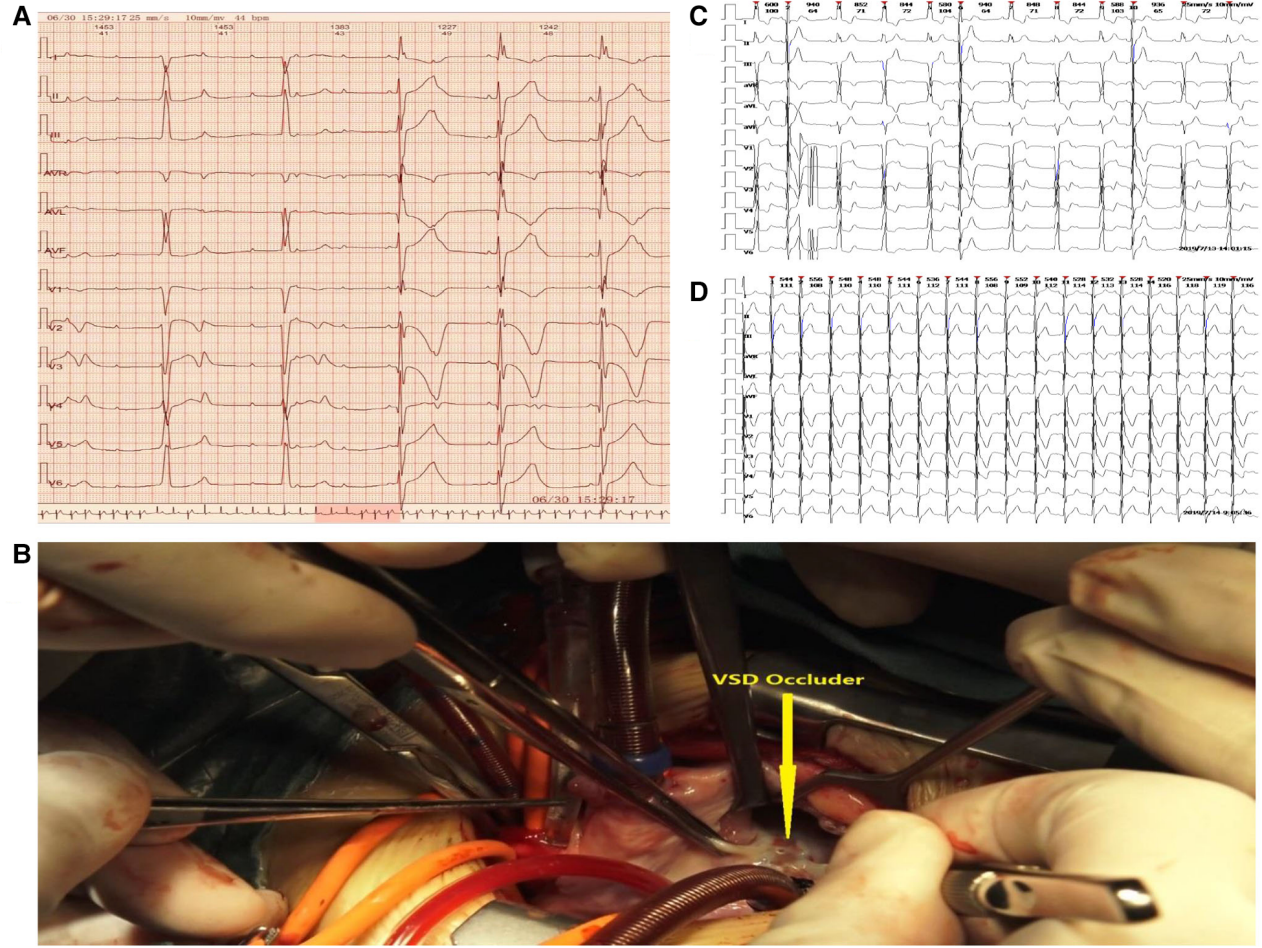
(**A**) Electrocardiogram of atrioventricular block before the surgery. (**B**) Identification and removal of the occluder. (**C**) Implantation of a temporary epicardial pacemaker and continuous pumping of isoproterenol. (**D**) Recovery of the heart rhythm.

In July 2019, the parents of the child noticed a significant decrease in their child's activity and visited the hospital for further examination. Electrocardiogram (Figure 1A) revealed sinus rhythm, left deviation of the electrical axis, third-degree atrioventricular block, and complete left bundle branch block, accompanied by ST-T changes. Methylprednisolone sodium succinate pulse therapy failed to improve the child's condition after admission.

## 3. Diagnostic assessment

Conventional treatment was discontinued after discussion with the parents, and a permanent pacemaker was implanted. Removal of the occluder and repair of the pmVSD were performed surgically. It was decided that a permanent pacemaker would be implanted if the function of the atrioventricular node (AV node) could not be restored. Under extracorporeal circulation, the occluder was opened under the tricuspid septal valve through a right atrial incision. The fibrous tissue wrapped around the occluder was carefully freed along the surface of the occluder umbrella to prevent damage to the surrounding interventricular septum and to remove the occluder completely. The diameter of the residual interventricular septal defect was 3 mm, and continuous suturing with a pericardial patch was performed (Figure 1B). The surgical procedure was uneventful. After the surgery, a temporary epicardial pacemaker was implanted, and isoproterenol was administered continuously (Figure 1C). The heart rhythm gradually recovered after 3 days (Figure 1D) and was maintained throughout the 45 months of follow-up.

## 4. Discussion

CAVB is a serious complication of transcatheter VSD closure, which may lead to Adams–Stokes syndrome and sudden death. Improved occluder devices with longer waists that exert a lower pressure on the tissue surrounding the defect have become available in recent years. Although the theoretical incidence of CAVB is low, it cannot be avoided completely (7). The reported incidence of CAVB after transcatheter closure of VSD is 1%–5% (8–10); however, a recent meta-analysis of transcatheter closure of VSD reported the incidence of CAVB as 0.8% (11). The mechanism of CAVB after transcatheter closure of VSD may be related to the occurrence of inflammatory edema around the defect (12). According to the study by Walsh et al., the atrioventricular block that occurs immediately after the placement of the occluder, which may be directly caused by mechanical compression, and the atrioventricular block that occurs within weeks and months after the placement of the occluder, which may be caused by inflammation and fibrosis, result in the occurrence of CAVB (13).

MITC has gradually developed into an effective alternative to traditional surgery for repairing pmVSD in recent years (14). Numerous studies have reported that the success rate of this surgery is comparable with that of percutaneous catheter occlusion and surgical repair assisted by extracorporeal circulation (15, 16). Some studies have suggested that MITC is a safe and effective choice for patients with pmVSD, even when performed as the first choice of treatment (16, 17). In China, MITC is considered an effective treatment for pmVSD. Compared with that of transcatheter closure, MITC requires a shorter path, which prevents friction of the conduction system and damage to the valve and surrounding tissue. Thus, inflammatory edema is less likely to occur postoperatively. In addition, MITC is simple and easy to repeat, the selection of the occluder is more individualized, and it is less likely to produce compression. According to a recent meta-analysis by Hong et al., the incidence of CAVB after MITC of VSD was 0.2%, with no incidence of delayed CAVB (3).

CAVB is a serious and difficult complication of pmVSD occlusion surgery. However, the management of CAVB remains controversial. Previous studies have reported that CAVB is prone to recurrence after arrhythmia occurs intraoperatively. It is recommended to terminate the surgery when arrhythmia occurs intraoperatively (13). The effectiveness of early postoperative CAVB steroid therapy has been established (8, 18), which may be related to a reduction in early inflammatory edema. Some studies have also reported the incidence of early CAVB after the removal of the occluder to restore heart rhythm (18, 19). However, few studies have reported the incidence of delayed-onset CAVB in patients who showed recovery of the heart rhythm through steroid therapy. Permanent pacemaker implantation is the primary treatment for delayed-onset CAVB at present (5, 6, 19). Lin et al. reported that the incidence of permanent CAVB after pmVSD and permanent pacemaker implantation occlusion were 0.7% and 0.5%, respectively. Removal of the device is an effective treatment for restoring normal conduction in patients with acute and subacute CAVB (6).

In the present case, CAVB occurred 2 years and 5 months after performing MITC of pmVSD. The incidence of CAVB after MITC of pmVSD is lower than that with transcatheter closure of pmVSD (1, 2). However, delayed CAVB may still occur in the long term after surgery, suggesting that the follow-up duration after MITC of pmVSD should be prolonged. It was found that the diameter of the residual interventricular septal defect decreased during the surgical removal of the occluder. Thus, it is possible that the defect shrinks during the growth and development of the child or that occluder stimulation leads to gradual fibrosis of the surrounding tissue. Chinese experts recommend that the patient should be over the age of 3 months at the time of MITC of VSD. However, we suggest that a more conservative age indication should be adopted since delayed CAVB may be caused by a smaller defect after growth of the patient and the larger compression area of the occluder. In the present case, the heart rhythm recovered after the removal of the occluder, which has been speculated to be an effective treatment for delayed CAVB. Xie et al. also reported on the resolution of long-term delayed CAVB after percutaneous transcatheter closure of an interventricular septal defect by removing the occluder after steroid treatment failure (20). Helping patients recover their autonomic rhythm is superior to implanting a permanent pacemaker. Thus, it is recommended to actively remove the occluder in patients with CAVB, and clinicians must strive to restore an autonomous heart rhythm in such patients.

In conclusion, MITC is successful in the treatment of pmVSD, with evident advantages. However, the incidence of delayed CAVB cannot be avoided. It is important to evaluate the safety of this procedure and follow stricter indications, particularly for young children, in addition to adopting more conservative age indications. The follow-up duration should be extended during the postoperative clinical management after MITC of pmVSD, and occluder removal is recommended in cases of postoperative CAVB.

## Data Availability

The original contributions presented in the study are included in the article, further inquiries can be directed to the corresponding author.
